# Dementia care from the perspective of family members, caregivers, and public health and social care professionals: a qualitative study of the Italian fund for Alzheimer’s and other dementias

**DOI:** 10.3389/fpubh.2025.1726733

**Published:** 2026-01-07

**Authors:** Annachiara Di Nolfi, Vittorio Palermo, Ilaria Palazzesi, Serena Passoni, Flaminia Camilli, Alice Paggetti, Antonio Ancidoni, Elisa Fabrizi, Patrizia Lorenzini, Guido Bellomo, Francesco Sciancalepore, Nicoletta Locuratolo, Paola Scardetta, Angela Giusti, Nicola Vanacore, Francesca Zambri

**Affiliations:** 1 National Center for Disease Prevention and Health Promotion, National Institute of Health (ISS), Rome, Italy; 2 Department of Biomedicine and Prevention, Universita degli Studi di Roma Tor Vergata, Rome, Italy; 3 Cognitive Neuropsychology Center, Azienda Socio Sanitaria Territoriale Grande Ospedale Metropolitano Niguarda, Milan, Italy; 4 Psychologist, Rome, Italy; 5 Escuela de Doctorado, Universidad Católica de Valencia San Vicente Mártir, Valencia, Spain; 6 Department of Human Neuroscience, Sapienza University of Rome, Rome, Italy

**Keywords:** dementia, caregiver, health and social care professionals, health needs, qualitative research, public health

## Abstract

**Background:**

Longer life expectancy has increased the prevalence of dementia, a major cause of disability in old age, requiring an interdisciplinary approach involving health and social care professionals (HScPs) and family members/caregivers (FmCs). This study aims to describe the current state of dementia care in Italy, identifying strengths, weaknesses, and experiences from FmCs and HScPs.

**Study design:**

Descriptive qualitative study.

**Methods:**

Forty-two focus groups have been conducted with 329 participants (187 HScPs and 142 FmCs).

**Results:**

The management of dementia is hampered by a marked unevenness in territorial services, with often insufficient services and staff. FmCs complain of difficulties in obtaining information and face a burden of care, exacerbated by the fragmentation of services and the COVID-19 pandemic. Despite these critical issues, the support of dementia-specific services (e.g., Centres for Cognitive Disorders and Dementias, Day Care Centres) and associations emerged as crucial. To enhance care, participants emphasized the need for more uniform and integrated services, well-trained professionals, public awareness campaigns to reduce stigma, and increased support for people living with dementia (PLWD) and their families.

**Conclusion:**

A holistic and coordinated approach that reduces territorial inequalities and empower effective resources is essential to ensure equitable care and improve the quality of life of PLWD and their families.

## Introduction

Life expectancy in developed countries has risen significantly due to advances in healthcare, social, and economic conditions (1, 2), but this has also increased the prevalence of diseases like dementia (3, 4). The number of people living with dementia (PLWD) is projected to double every 20 years, reaching 75 million by 2030 and 135 million by 2050 (5), making it one of the most widespread neurodegenerative diseases (6) and a leading cause of disability in older adults. Caring for PLWD requires a multidisciplinary and interdisciplinary approach involving health and social care professionals (HScPs) and family members/caregivers (FmCs). As PLWD lose self-sufficiency, caregiver responsibilities intensify (6–8), leading to physical, emotional, and financial burdens, collectively known as “*caregiver burden*” (9). Dementia also impacts work productivity (10), showing its societal implications beyond individuals. An interdisciplinary and cross-sectoral approach is essential for achieving early diagnosis and treatment, which can help the patient and caregiver to reduce medical and disease-related consequences, improve the quality of life, and optimize the use of available resources (11).

Dementia has a significant impact not only on FmCs, but also on clinicians and researchers, who must navigate ethical and legal challenges that evolve with the stage and severity of the disease (12). These challenges often involve diagnosis, treatment and care decisions, including the management of artificial nutrition and hydration at the end of life (13). Inadequate management of PLWD can exacerbate cognitive and non-cognitive symptoms, further increasing the burden on both formal and informal caregivers (14).

Care for migrants with dementia in Italy also faces significant challenges. The main difficulties involve linguistic and cultural barriers, diagnostic complexity, and the involvement of FmCs in care pathways (15). Despite this, many services report growing attention to this population, recognizing their clinical and social needs as increasingly important. Some aspects, such as the management of pharmacological treatments and neuropsychiatric symptoms, are less problematic. Ensuring inclusive care pathways that are sensitive to cultural diversity remains a priority (16).

For these reasons, the care burden also become a significant concern for HScPs involved in the care of people with disabilities, particularly in managing Behavioural and Psychological Symptoms of Dementia (BPSD) (17).

COVID-19-related restrictions implemented to manage the pandemic have exacerbated these vulnerabilities (18, 19). For more than 2 years, PLWD and their FmCs were required to isolate themselves from support systems, change routines and reduce their use of services (20, 21), some of which underwent significant reorganisation (22, 23).

With this context, the Italian Fund for Alzheimer’s and other Dementias (IFAD) (24, 25) operates as one of the largest public health initiatives addressing dementia in Italy. This qualitative study is part of that initiative.

The study aimed to describe the state of care provision for PLWD in Italy, focusing on strengths, weaknesses, and experiences from the perspectives of FmCs and HScPs.

## Methods

A descriptive qualitative study was conducted (26). Two focus groups (FGs) were organized in each Italian Region and Autonomous Provinces: one with FmCs and the other with HScPs (e.g., psychologists, nurses, geriatricians, neurologists, and social workers). Participants were recruited through purposeful sampling, selecting HScPs and FmCs actively involved in dementia care at the time of the study. All participants were contacted by an IFAD collaborator through the project’s stakeholder network, including regional dementia representatives and national families and patients’ associations. This initial contact was followed by an email invitation.

Due to the large number and in order to facilitate the organisation and conduct of the FGs, they were carried out both online via the Italian National Institute of Health (INIH) platform and in person at healthcare facilities or at the offices of family members’ and patients’ associations. Experienced researchers from INIH (authors FZ, ADN, VP, AG, SP, IP) facilitated the FGs using a semi-structured question guide tailored to the target participants (Table 1).

**Table 1:** List of aims and questions for the focus group.

**Aim 1.** Strengths and weaknesses of regional/provincial care pathways
**(1a)** In your opinion, what are currently the strengths and weaknesses of the care of PLWD in your Region/Autonomous Provinces? The discussion can be on the perspective of: the available diagnostic tools (e.g., cross-cultural cognitive assessment tools) – *Only for professionals* communication of diagnosis pharmacological treatment **(1b)** Concerning the provision of non-pharmacological treatments (e.g., living environment interventions, social interventions, also linked to the culture of origin), what are, in your opinion, the critical issues and opportunities? **(1c)** Thinking about caregiver care, what works and what does not work in your Region/Autonomous Provinces? **(1d)** What about the caring of professionals? What works and what does not work in your Region/Autonomous Provinces? – *Only for professionals*
**Aim 2.** Areas of improvement (in care pathways, communities, policies, etc.)
**(2)** In your opinion, what are the possible areas for improvement both in care pathways and in communities and policies?
**Aim 3.** Impact of the COVID-19 emergency on the provision of care for PLWD and their caregivers
**(3a)** In your opinion, what have been the effects of the COVID-19 pandemic on the provision of care and care pathways of PLWD and their caregivers? **(3b)** How have services responded to the emergency?
**Aim 4.** Training needs of family members, caregivers and health and social care professionals
**(4a)** In your opinion, what are the training needs of family members/caregivers caring for PLWD? – *Only for family members/caregiver* **(4b)** In your opinion, what are the training needs of professionals involved in caring of PLWD? – *Only for professionals*

PLWD, people living with dementia.

Each FG lasted approximately 90 min, with a brief researchers’ introduction on the study’s aims and context. They were audio-recorded, and transcribed. Socio-demographic data were collected anonymously. Three authors (FZ, ADN, VP) independently read and coded the transcripts, with a categorial analysis. Subsequently, the authors met to standardize the individual codings and resolve discrepancies through discussion. Most categories were predefined based on the main research questions (deductive approach), while further categories were identified as they emerged during the coding process (inductive approach). Furthermore, the researchers compared and analysed the qualitative data by clustering them according to emerging categories and organizing them by each participating Region, as detailed in Supplementary materials 1, 2. NVivo software was used for the qualitative analysis. Data saturation was not required. The tree-nodes were applied to all transcripts and the most meaningful verbatim were identified. The quality of reporting was assessed using Consolidated Criteria for Reporting Qualitative Research (COREQ) (27), as detailed in Supplementary materials 3. The *Research team and reflexivity* domain of the COREQ checklist is partially fulfilled, as some information regarding the interviewer’s role and characteristics was not fully reported. The *Study design* domain is largely fulfilled, with a good description of sampling, data collection, and methodological approach, although with some minor omissions. The *Analysis and findings* domain is fully satisfied, through a clear description of coding procedures, theme development, software use, and consistency between data and findings.

## Results

Between October 2022 and July 2023, 42 FGs were conducted: 21 with FmCs and 21 with HScPs, involving 329 participants (187 HScPs and 142 FmCs). In the study sample, most caregivers were female and family members (primarily daughters and wives), while male caregivers and non-family caregivers (e.g., friends, formal caregiver, volunteers) were underrepresented. Participant characteristics are detailed in Table 2.

**Table 2:** Characteristics of participants involved in the FGs.

Data of participants’	N
Participants	
Health and social care professionals	187
Family members/caregiver	142
Median age in years	52 (IQR 43–61)
Gender	
Women	243
Men	86
Health and social care professionals (*n* = 187)	
Psychologist	39
Nurse	27
Geriatrician	26
Social-health operator	22
Neurologist	19
Occupational therapist	11
Social worker	10
Physiotherapist	8
Professional educator	7
Speech therapist	5
General practitioner	3
Psychiatrist	3
Psychiatric rehabilitation technician	2
Health visitor	1
Social facilitator	1
Nuclear medicine physician	1
Music therapist	1
Project Manager	1
Family members/caregiver (*n* = 142)	
Daughter/Son	88
Wife	27
Husband	13
Daughter-in-law	5
Sister	4
Nephew	3
Sister-in-law	1
Father	1

The main themes explored were:

- Strengths and weaknesses of regional/provincial care pathways for PLWD;
- Improvements in care provision;
- The impact of COVID-19 emergency on the care provision for PLWD and their FmCs;
- Training needs of both FmCs and HScPs.

The results are presented below; detailed findings specific to HScPs and FmCs are provided in Supplementary materials 1 and 2.

### The strengths and weaknesses of regional/provincial care pathways for PLWD

The availability of specialized services for PLWD, such as Centres for Cognitive Disorders and Dementias (CCDDs), Day Care Centres (DCCs), and Nursing Homes (NHs), is a key strength identified by both HScPs and FmCs. Certain regions reap considerable benefits from a well-established network that integrates health services with local resources, such as social work, home care, and community associations.

*HScPs: “…we have a territory that works in a capillary way. We have these CCDDs that are well distributed throughout the territory to reach the patient and the family member... (...)”*

Some CCDDs provide psychological support and training courses for FmCs to improve their ability to cope with the disease. Additionally, some CCDDs offer tailored training for HcSPs to keep them updated on dementia care.

*HScPs: “...another thing that I see working very well is the (...) multi-professional part of the training, which is aimed at both the family assistants and the FmCs.”*

The HScPs highlighted the professionalism, sensitivity, and teamwork of the staff as key strengths, often enhanced by interdisciplinary collaboration and supported by dedicated research initiatives or projects in certain contexts.

*HScPs: “...among colleagues, we confront each other in critical or special situations; therefore (...) there is a good climate within the team, which allows us to deal with any difficulties that may arise...”*

Dementia associations are a key strength in many Regions, offering support to FmCs through training activities and Alzheimer Cafés (AC). These initiatives help families manage daily life with PLWD and encourage experience sharing.

*FmCs: “(…) it [Alzheimer Café] allows the creation of that system of connections between caregivers and between patients, where one no longer feels so alone (...). These are all moments that (...) make everyday life less difficult to cope with”*

DCCs play a fundamental role, particularly in providing non-pharmacological treatments that enhance the well-being of patients and families. Additionally, some communities demonstrate exemplary practice by working in synergy with local associations and institutions, to promote inclusion, raise awareness, and provide support through initiatives such as Dementia Friendly Communities (DFCs). A DFC is defined as a space characterized by accessibility, inclusion, and the empowerment of PLWD and their families, requiring responsive organizations, early diagnosis, and the implementation of social capital for collective action (28, 29).

*FmCs: “Dementia Friendly Communities have been created, (...) so there are services (...) and a working group (...): there are trainings on the territory, caregivers’ groups, there is cognitive stimulation, there is really a lot...”*

Despite these positive aspects, service distribution is not uniform, with CCDDs, CCDs, and NHs unevenly spread across regions, often understaffed and not inclusive of all professionals.

*HScPs: “...there are 21 different regional health systems and, paradoxically, almost all of them have a way of defining even the same type of service [in a] different way; (...)”*

Both FmCs and HcSPs reported that DCCs and NHs have limited capacity and long waiting lists, leaving many without care and forcing families to rely on expensive private services.

*FmCs: “The sore point is the waiting lists, there is the impossibility of accessing these facilities [ed. DCCs] (...) the activities [ed. non-pharmacological treatment] are effective in the early stages of the disease... (...)”*

*FmCs: “(…). This means that some require a completely private supply and then it is up to the user to pay the full fee...”*

Legislative and bureaucratic processes present significant challenges. FmCs reported that lengthy and complex procedures for disease recognition and financial support hinder timely care for PLWD.

*FmCs: “...then it is not enough to help our parents, but there is the whole bureaucratic part, which is a continuous preparation of documents, going to social services, going to the municipality, going to the doctor. (...) I have to submit a document several times to the same office...”*

FmCs reported challenges in managing drug treatments, with frequent adjustments requested by relatives, and noted that non-pharmacological treatments are not consistently available across all settings.

*FmCs: “I was the one who had to say to the doctor: “Maybe it’s better to lower it to bring the parameters back a little bit”...”*

*FmCs: “I didn’t have any offer [of non-pharmacological therapies] and everything I do I do it myself, I look for things, I read, I ask the association, I confront the association’s psychologist…”*

FmCs expressed that they frequently receive crucial information about the disease but lack emotional support and clear guidance on how to access available services. The absence of proper service mapping contributes to their feelings of isolation when facing tough decisions and managing the disease.

*FmCs: “There is no reference, there is no information, there is nothing. What I can do is look for information, open the computer and search on the Internet, but it’s not a good way to go, it’s very confusing. (...)”*

HcSPs often face challenges in supporting PLWD due to a lack of psychological support and an overwhelming workload. The inadequate staff-to-workload ratio and precarious contracts hinder continuity of care. Home care is seen as essential but insufficient, with care responses being fragmented due to complex bureaucracy, poor coordination between professionals, lack of stable points of contacts, and inconsistent service provision. This fragmentation leads to confusion and isolation for both patients and carers.

*HcSPs: “‘...our precariousness does not allow us to plan in the long term... a project of this kind would need a time perspective, even our own stability within this service, (...)”*

Both FmCs and HcSPs highlighted that general practitioners (GPs) often do not provide sufficient support to PLWD and their families. This is due to gaps in training on disease management and a lack of knowledge or networking with CCDDs and DCCs. In some Regions, the issue is worsened by the absence or poor implementation of integrated care pathways (ICPs) for dementia.

*HcSPs: “...we find it difficult to cooperate with GPs, both in requesting visits and in following up the patient. (…), there are family members of patients who turn to us specialists because the GP sends them directly to us...”*

Difficulties arise in non-specialized services like emergency departments, long-term care units, and other non-specialist facilities, where insufficient staff training hampers proper care for PLWD, causing additional distress. Family assistants also face challenges due to a lack of training in dementia care.

*FmCs: “...when a PLWD goes to hospital, they are not cared for at all. (…), the doctor who attended him [the father] gave him a discharge form because my father was not a cooperative person”*

A key issue for FmCs is the lack of a structured support network, making caregiving highly demanding and affecting their quality of life and well-being. Social isolation is common, worsened by challenges in managing the PLWD’s social interactions as the disease progresses, as well as by the stigma surrounding the disease in many communities.

*FmCs: “I left my job, I left my house, I left everything, I left my life in a nutshell. (...) I don’t even have my own room here [ed. at his parents’ home], (...). I don’t know how much longer I can do this”*

### Improvements in care provision

Both FmCs and HcSPs emphasized the need to increase the number of CCDDs and DCCs and improve the multidisciplinary nature of professional teams, including better networking with GPs. They also highlighted the importance of including professionals often underrepresented, such as psychologists, occupational therapists, and social workers. The development and implementation of regional ICPs was suggested as a solution to address disparities in care provision.

*HcSPs: “[It is necessary] to create a dedicated ICP with dedicated outpatient activities, trained staff who take charge together with all the other figures to follow the patient.”*

For the residential and semi-residential sector, there was a focus on strengthening Alzheimer Units and promoting personalized interventions for PLWD. Greater access to non-pharmacological treatments within DCCs was identified as a key area for improvement.

*HcSPs: “[We should] (…) offer a personalisation of rehabilitation; identify rehabilitations targeted at the person, (...) it becomes difficult for an older adult to reach certain areas; (…)”*

There is a need for training in managing the diagnostic process, along with practical guidance on next steps and available resources. Additionally, psychological and educational support for both FmCs and HcSPs was highlighted as a crucial area for improvement.

*FmCs: “(...). We need to be informed about what dementia is and how best to communicate with and manage people with dementia, (…).”*

Finally, simplifying bureaucratic procedures for disease recognition and implementing awareness initiatives on dementia, supported by the third sector, were recommended to reduce stigma and promote social inclusion. The lack of specific training for HcSPs leads to challenges in managing PLWD in non-specialist services like emergency departments. Establishing priority pathways and awareness programs is suggested to improve reception and treatment. Additionally, training and information should extend beyond professionals and family assistants to include communities, with efforts to create new Dementia Friendly Communities.

### The impact of COVID-19 emergency on the provision of care for PLWD and their FmCs

The COVID-19 pandemic exacerbated challenges for PLWD, worsening cognitive and behavioral conditions due to social isolation. Service disruptions and limited alternatives increased the burden on family carers, while reduced visits to NHs intensified loneliness.

*HcSPs: “The day centres really did close in the middle of the pandemic (...) for several months, so suddenly the routine of the users themselves was completely changed... so it was not just a return home, but an isolation...”*

Fear of infection further decreased service use and delayed care. However, the crisis prompted service reorganization and the adoption of telemedicine, offering a valuable complement to traditional care.

*HcSPs: “...we have used telemedicine a lot, both in the Covid period and in the post-Covid period; in fact, we have televisita, which was equated by our Region with a control visit.”*

### The training needs of FmCs and HScPs

HScPs emphasized the need for training on different dementia subtypes, symptoms, treatments (including non-pharmacological options), and multidisciplinary care. Specific training for GPs was recommended to improve early diagnosis and referrals, along with communication skills for sensitive situations. Priorities also included behavioral management, palliative care, and ethical considerations, alongside better awareness of local support networks. FmCs highlighted the need for training in dementia basics, caregiving strategies, and coping mechanisms for stress and isolation. They also stressed the importance of practical guidance for navigating services, accessing resources, and addressing bureaucratic and legal challenges.

## Discussion

The study reveals that dementia management is a complex issue involving multiple stakeholders, with CCDDs and DCCs providing essential support despite their limited availability. However, public awareness remains low, increasing stigma and isolation for PLWD and families. Dementia-Friendly Communities and association-led initiatives help improve inclusion. Non-specialist HScPs, particularly in long-term care and emergency settings, require targeted training, while inadequate GP support in referrals remains a challenge.

The heterogeneity of the distribution and organization of services, with significant regional disparities, creates disorientation for families. Both FmCs and HScPs reported fragmented care due to insufficient and precarious staff dedicated to dementia care, particularly at the local level. This leads many FmCs to turn to private services, more expensive, exacerbating health inequalities, especially in regions with limited access to public care and long waiting lists.

Establishing regional ICPs is crucial to reducing inequalities and care fragmentation. Enhancing training for HScPs and FmCs can improve dementia management, while simplifying bureaucracy would facilitate access to home services and benefits. Moreover, collaboration between health sector with the third sector could enhance territorial service provision, empowering existing resources. The pandemic exposed systemic weaknesses but also drove innovation, leading to the lasting integration of telemedicine into traditional care.

The study confirms the significant economic impact of dementia on families and society, with indirect costs—mainly unpaid care—accounting for 75% of total expenses. Lost productivity further amplifies the financial burden (30, 31). Additionally, beyond diagnosis and treatment, substantial costs arise from home care and continuous family support, as PLWD often require round-the-clock assistance early in the disease (32, 33).

The study sample reflects the predominance of female family caregivers, as also confirmed in other Italian studies on dementia care (34). However, male caregivers and formal caregivers were only marginally represented. This limited diversity may constrain the transferability of some findings, particularly regarding the different caregiving roles, support needs, and care dynamics that characterize male or formal caregivers. Preventive and health promotion strategies are crucial, as dementia is not an inevitable outcome of aging. Lifestyle factors, including physical inactivity, tobacco use, poor diet, harmful alcohol consumption, social isolation, and cognitive inactivity, make dementia prevention possible through public health interventions (35). Furthermore, caregiver and community support enhance well-being of PLWD by providing coping strategies (36). In Italy, IFAD has facilitated health promotion and prevention activities through the collaboration between the National Institute of Health and the Local Authorities. Special attention is needed for disadvantaged social groups (including those with a migration background), who face from 30 to 65% higher risk of chronic diseases, including dementia (37–39).

This study highlights the value of using qualitative tools to understand the needs and expectations of FmCs of PLWD and HScPs. The diverse perspectives of participants enriched the understanding of potential solutions and areas for improvement. Key findings emphasize the need to strengthen service networks, starting with the reinforcement of primary health care, to improve coordination among structures, and increase human and financial resources across Italian regions and Autonomous Provinces. Raising public awareness, also among personnel working in non-specialized services, is critical for fostering a culture of inclusion and social solidarity. An integrated approach that addresses the needs of PLWD while prioritizing the well-being of FmCs and HScPs is essential for significantly improving the quality of life for all involved.

A holistic and coordinated approach to dementia care, involving all stakeholders and addressing the needs of families, is crucial for equitable and sustainable care. Healthcare practices and policies in Italy must respond to the challenges of an aging population and increasing care complexity. The Italian National Dementia Plan (40) provides a structured strategy to improve diagnosis, patient care, and family support, while the new Italian Alzheimer’s and Dementia Fund (2024–2026) (41) represents a concrete investment to strengthen the integrated network of services, promote professional training, and reduce regional inequalities. These national efforts align with the WHO Global Action Plan on the Public Health Response to Dementia and European strategies aimed at improving dementia care across countries (42). Ensuring the synergy of these initiatives is essential to prevent any patient or family from being left isolated. Investing in awareness campaigns, telemedicine, and institutional coordination remains key to achieving a more inclusive and equitable healthcare system.

## Limitations

Participants’ recruitment through services and associations networks, excluded individuals outside these circles, limiting diverse perspectives, especially among FmCs. Male caregivers and formal caregivers were underrepresented, limiting the diversity of perspectives included. Additionally, participants were not recruited from all Italian provinces, focusing findings on areas with more developed services. In the FGs of some Regions/Autonomous provinces only some professional figures participated, limiting the reading of the phenomenon. The absence of FmCs of migrants and key professionals like cultural mediators did not bring out the perspectives of this specific group.

## Data Availability

The raw data supporting the conclusions of this article will be made available by the authors upon justified request.
