# Supplementary material for: Dementia care from the perspective of family members, caregivers, and public health and social care professionals: a qualitative study of the Italian fund for Alzheimer’s and other dementias
Source: Front Public Health. 2026 Jan 7;13:1726733. doi: 10.3389/fpubh.2025.1726733 (PMC12819221; doi:10.3389/fpubh.2025.1726733)
Supplement: Supplementary file 1 [file Data_Sheet_1.pdf]

**Consolidated criteria for reporting qualitative studies (COREQ): 32-item checklist (Tong et al. 2007)**

| No. | Item | Description | Section # |
|---|---|---|---|
| **Domain 1: Research team and reflexivity** | | | |
| Personal characteristics | | | |
| 1. | Interviewer/facilitator | *Which author/s conducted the interview or focus group?* | Authors' contributions |
| 2. | Credentials | *What were the researcher's credentials?* | Title page |
| 3. | Occupation | *What was their occupation at the time of the study?* | / |
| 4. | Gender | *Was the researcher male or female*? | / |
| 5. | Experience and training | *What experience or training did the researcher have?* | Methods |
| Relationship with participants | | | |
| 6. | Relationship established | *Was a relationship established prior to study commencement?* | Methods |
| 7. | Participant knowledge of the interviewer | *What did the participants know about the researcher? E.g. Personal goals, reasons for doing the research* | Methods |
| 8. | Interviewer characteristics | *What characteristics were reported about the interviewer/facilitator? E.g. Bias, assumptions, reasons and interests in the research topic* | / |
| **Domain 2: Study design** | | | |
| Theoretical framework | | | |
| 9. | Methodological orientation and theory | *What methodological orientation was stated to underpin the study? E.g. grounded theory, discourse analysis, ethnography, phenomenology, content analysis* | Methods |
| Participant selection | | | |
| 10. | Sampling | *How were participants selected? E.g. purposive, convenience, consecutive, snowball* | Methods |
| 11. | Method of approach | *How were participants approached? E.g. face-to-face, telephone, mail, email* | Methods |
| 12. | Sample size | *How many participants were in the study*? | Results/Table 3 |
| 13. | Non-participation | *How many people refused to participate or dropped out? What were the reasons for this?* | / |
| Setting | | | |
| 14. | Setting of data collection | *Where was the data collected? E.g. home, clinic, workplace* | Methods |
| 15. | Presence of non-participants | *Was anyone else present besides the participants and researchers?* | / |
| 16. | Description of sample | *What are the important characteristics of the sample? E.g. demographic data, date* | Results/Table 3 |
| Data collection | | | |

| | | | |
|---|---|---|---|
| 17. | Interview guide | *Were questions, prompts, guides provided by the authors?*<br>*Was it pilot tested?* | Methods/Table 1 |
| 18. | Repeat interviews | *Were repeat interviews carried out? If yes, how many?* | / |
| 19. | Audio/visual recording | *Did the research use audio or visual recording to collect the data?* | Methods |
| 20. | Field notes | *Were field notes made during and/or after the interview or focus group?* | / |
| 21. | Duration | *What was the duration of the interviews or focus group?* | Methods |
| 22. | Data saturation | *Was data saturation discussed?* | Methods |
| 23. | Transcripts returned | *Were transcripts returned to participants for comment and/or correction*? | / |
| **Domain 3: analysis and findings** | | | |
| Data analysis | | | |
| 24. | Number of data coders | *How many data coders coded the data?* | Methods/Authors' contributions |
| 25. | Description of the coding tree | *Did authors provide a description of the coding tree?* | Methods/Tables 2-3 |
| 26. | Derivation of themes | *Were themes identified in advance or derived from the data?* | Methods |
| 27. | Software | *What software, if applicable, was used to manage the data?* | Methods |
| 28. | Participant checking | *Did participants provide feedback on the findings?* | / |
| Reporting | | | |
| 29. | Quotations presented | *Were participant quotations presented to illustrate the themes/findings?*<br>*Was each quotation identified? E.g. Participant number* | Results<br>/ |
| 30. | Data and findings consistent | *Was there consistency between the data presented and the findings?* | Results |
| 31. | Clarity of major themes | *Were major themes clearly presented in the findings?* | Results |
| 32. | Clarity of minor themes | *Is there a description of diverse cases or discussion of minor themes*? | Results |

Allison Tong and others, Consolidated criteria for reporting qualitative research (COREQ): a 32-item checklist for interviews and focus groups, International Journal for Quality in Health Care, Volume 19, Issue 6, December 2007, Pages 349–357, https://doi.org/10.1093/intqhc/mzm042
