# Supplementary material for: Dementia care from the perspective of family members, caregivers, and public health and social care professionals: a qualitative study of the Italian fund for Alzheimer’s and other dementias
Source: Front Public Health. 2026 Jan 7;13:1726733. doi: 10.3389/fpubh.2025.1726733 (PMC12819221; doi:10.3389/fpubh.2025.1726733)
Supplement: Supplementary file 2 [file Data_Sheet_2.pdf]

Supplementary Material 2 - Emerging categories reported by HScPs

	Abruzzo	Basilicata	Calabria	Campania	Emilia-Romagna	Friuli Venezia Giulia	Lazio	Liguria	Lombardia	Marche	Molise	Piemonte	Puglia	Sardegna	Sicilia	Toscana	Umbria	Val d'Aosta	Veneto	PA Trento	PA Bolzano
**Strengths**																					
Functioning of dedicated services		✓			✓			✓			✓		✓			✓				✓	
– Availability and functioning of DCCs				✓						✓	✓		✓			✓	✓			✓	
– Functioning of CCDDs					✓			✓		✓	✓			✓	✓			✓		✓	
– Availability and functioning of residential care					✓		✓		✓				✓	✓	✓		✓				✓
– Functioning of home care			✓	✓	✓		✓									✓			✓	✓	✓
– Functioning of social services					✓							✓						✓			
Networking between services			✓		✓			✓		✓		✓					✓		✓	✓	✓
Collaboration with GPs			✓														✓		✓		✓
Multidisciplinary and team-based care		✓	✓		✓		✓	✓	✓	✓	✓	✓			✓		✓	✓	✓	✓	✓
– Discussion of cases							✓	✓	✓	✓	✓					✓					
HScPs sensitivity and commitment	✓		✓	✓			✓	✓		✓	✓						✓				✓
– Willingness to network							✓	✓		✓	✓										
Taking care of FmCs		✓					✓	✓	✓	✓	✓	✓			✓		✓	✓		✓	✓
– Provision of education and training initiatives					✓		✓	✓		✓	✓						✓			✓	✓
– Provision of psychological support					✓		✓	✓		✓						✓	✓			✓	✓
Taking care of HScPs										✓											
– Provision of training initiatives					✓			✓			✓							✓		✓	
Presence of associations and third sector initiatives			✓	✓	✓	✓		✓	✓	✓	✓	✓	✓				✓	✓		✓	✓
– Organisation of AC			✓																		
Availability of non-pharmacological treatments					✓	✓	✓	✓			✓		✓	✓	✓		✓	✓	✓	✓	✓
Presence of dementia research activities and projects	✓								✓									✓		✓	
**Weaknesses**																					
Organisation and functioning of CCDDs	✓		✓	✓						✓		✓	✓	✓	✓	✓					
– Reduced psychological care					✓					✓						✓					
– Absence of certain HScPs in CDCDs (occupational therapists, psychologists, etc.)					✓					✓									✓	✓	
Reduced availability of DCCs	✓		✓	✓			✓	✓					✓	✓	✓			✓			✓
Functioning of home care		✓		✓	✓		✓					✓	✓	✓	✓	✓	✓	✓		✓	✓
Functioning of psychosocial services		✓	✓		✓			✓		✓								✓		✓	
Availability and functioning of residential care							✓				✓			✓			✓	✓			
Heterogeneity of services			✓		✓	✓		✓		✓	✓	✓	✓	✓		✓	✓	✓	✓	✓	
Lack of integration of services and fragmentation of care	✓	✓	✓	✓	✓	✓	✓			✓	✓	✓	✓	✓	✓	✓	✓	✓	✓		
Lack of support and network with GPs			✓			✓	✓	✓	✓	✓	✓		✓	✓	✓			✓	✓	✓	✓
Reduced professional resources	✓	✓	✓		✓	✓	✓	✓	✓	✓	✓	✓	✓	✓	✓	✓	✓		✓	✓	✓
Lack/limited use of ICPs				✓						✓	✓									✓	
Long and fragmented diagnostic process			✓			✓					✓		✓	✓	✓	✓		✓		✓	
Lack of mapping of services					✓		✓		✓			✓	✓	✓	✓	✓				✓	✓
Long waiting lists	✓	✓	✓	✓	✓		✓		✓		✓	✓		✓	✓	✓	✓				✓
Limited places in facilities/centres		✓			✓		✓		✓	✓	✓							✓		✓	✓
Difficulties in managing pharmacological treatments					✓	✓			✓					✓							
Reduced provision of non-pharmacological treatments	✓		✓	✓	✓	✓			✓		✓		✓	✓							
Temporariness of Alzheimer's units and project-based services		✓														✓	✓			✓	

Lack of specialised facilities for people with early onset dementia					✓															✓	✓
Economic implications of private services	✓			✓					✓					✓				✓			✓
Reduced care of HScPs			✓	✓						✓			✓			✓		✓			
Reduced HScPs training	✓		✓		✓	✓	✓		✓	✓			✓		✓	✓					✓
– Reduced staff training in non-specialist services (e.g. E.R, long-term care facilities)							✓		✓		✓					✓	✓	✓	✓		✓
Reduced family assistants training					✓													✓			✓
Riduced care of FmCs	✓	✓	✓	✓			✓	✓	✓	✓	✓	✓		✓	✓	✓				✓	
Ineffective communication with family	✓	✓									✓		✓	✓				✓		✓	
– Ineffective communication of diagnosis					✓		✓						✓			✓					
Reduced disease knowledge and stigma			✓	✓	✓	✓			✓		✓	✓		✓	✓		✓	✓	✓		
**Improvements in care provision**																					
Improving dementia care services	✓	✓			✓								✓	✓		✓	✓	✓			✓
– Service networking and teamwork	✓	✓	✓	✓	✓			✓					✓		✓	✓	✓	✓			✓
– Strengthening psychological support for FmCs	✓	✓							✓					✓			✓			✓	
– Establishment of a FmCs information desk/system and mapping of services	✓	✓		✓		✓	✓	✓	✓				✓	✓		✓	✓			✓	
– FmCs training initiatives	✓	✓	✓			✓				✓					✓						✓
– Strengthening non-pharmacological treatments	✓			✓		✓	✓						✓								
– Strengthening home care		✓				✓											✓				
DCCS/CCDDs	✓												✓								
– Strengthening third sector activities					✓								✓		✓	✓		✓			
Increase in dementia care personnel						✓											✓	✓			✓
ICPs application										✓								✓			
Support and networking with GPs					✓			✓	✓				✓			✓				✓	
Training of HScPs		✓	✓			✓			✓				✓	✓	✓	✓			✓		✓
Strengthening/creating a psychological service for HScPs		✓																		✓	
Effective communication of diagnosis								✓												✓	
Increased financial support for families									✓												
Creating Dementia Friendly Communities						✓	✓						✓								
**The impact of COVID-19 emergency**																					
Closure of services/interruption of activities		✓	✓	✓		✓	✓		✓	✓	✓	✓	✓	✓	✓	✓	✓	✓	✓	✓	✓
Clinical deterioration of the patient	✓	✓	✓	✓	✓		✓		✓		✓	✓	✓	✓	✓	✓		✓	✓	✓	✓
Difficulties in managing the patient at home			✓																		✓
Reduction in visits to residential facilities			✓	✓	✓		✓		✓							✓		✓		✓	
Social isolation/need for socialisation	✓	✓	✓	✓			✓		✓						✓	✓			✓		✓
Fear of contagion affecting use of services	✓	✓		✓							✓				✓			✓			
Difficulty using telemedicine							✓		✓				✓				✓				
Delays in resuming activities		✓					✓		✓					✓	✓			✓			
Capacity for reorganisation					✓				✓	✓			✓		✓	✓	✓	✓	✓	✓	
Implementation of telecare/telemedicine	✓	✓	✓		✓			✓	✓	✓	✓	✓	✓	✓	✓	✓	✓		✓	✓	✓
**HScPs training needs**																					
Use of the integrated approach		✓	✓	✓			✓				✓	✓	✓		✓			✓		✓	✓
Classification and symptoms of dementia	✓	✓			✓					✓		✓									
Communicating with PLWD and FmCs		✓			✓				✓				✓			✓	✓	✓	✓		✓
Establishing a regional ICPs	✓		✓							✓	✓							✓			
Medications and dementia updates	✓									✓	✓	✓				✓				✓	
Management of behavioural problems	✓	✓			✓	✓	✓		✓	✓		✓			✓	✓	✓			✓	
Management of complications	✓															✓					
Palliative care and ethical issues	✓														✓	✓		✓			

Diagnostic techniques and their formulation	✓							✓		✓	✓		✓				✓			✓	
Non-pharmacological treatment updates	✓			✓		✓							✓		✓	✓					✓
