# Supplementary material for: Dementia care from the perspective of family members, caregivers, and public health and social care professionals: a qualitative study of the Italian fund for Alzheimer’s and other dementias
Source: Front Public Health. 2026 Jan 7;13:1726733. doi: 10.3389/fpubh.2025.1726733 (PMC12819221; doi:10.3389/fpubh.2025.1726733)
Supplement: Supplementary file 3 [file Data_Sheet_3.pdf]

Supplementary Material 3 - Emerging categories reported by FmC																					
	Abruzzo	Basilicata	Calabria	Campania	Emilia Romagna	Friuli Venezia Giulia	Lazio	Liguria	Lombardia	Marche	Molise	Piemonte	Puglia	Sardegna	Sicilia	Toscana	Umbria	Val d'Aosta	Veneto	PA Trento	PA Bolzano
**Strengths**																					
Functioning of health services (DCCs, CCDDs, NHs)	✓	✓	✓	✓	✓	✓		✓		✓	✓	✓		✓	✓	✓	✓	✓	✓	✓	
Functioning of home care	✓				✓	✓	✓													✓	✓
Reception by social services					✓									✓		✓				✓	
Provision of non-pharmacological treatments			✓			✓	✓	✓				✓					✓	✓		✓	
Support from GPs					✓	✓											✓			✓	
Multidisciplinary care				✓	✓				✓											✓	
Effective diagnostic process and communication of diagnosis	✓				✓															✓	✓
Availability of support with bureaucratic aspects									✓					✓							
Availability of third sector associations and initiatives					✓	✓	✓	✓	✓	✓		✓	✓	✓				✓	✓		✓
– Provision of psychological support for FmCs and sharing of experiences			✓		✓				✓	✓		✓	✓				✓	✓	✓		✓
– Provision of training and information support				✓	✓	✓		✓		✓	✓	✓	✓					✓	✓		
– Organisation of AC			✓				✓			✓		✓					✓				
Presence of Dementia Friendly Communities												✓									
**Weaknesses**																					
Functioning of health services	✓		✓	✓		✓															
– Functioning of outpatient services			✓																		
– Provision of DCCs	✓			✓							✓					✓		✓			
– Functioning of residential services		✓	✓		✓	✓		✓	✓	✓		✓	✓			✓	✓	✓			
– Availability and functioning of home care		✓	✓	✓						✓					✓	✓	✓	✓			
– Availability of places in facilities/centres						✓	✓									✓				✓	✓
– Reduced professional resources		✓	✓		✓	✓		✓	✓	✓	✓	✓						✓			
Functioning of social services					✓																
Lack of networking between services			✓					✓		✓											
Poor territorial distribution of associations, AC and DCCs		✓	✓	✓			✓	✓		✓	✓	✓	✓	✓	✓						✓
Long waiting lists for access to services/facilities	✓	✓	✓	✓	✓	✓			✓	✓	✓		✓	✓		✓	✓			✓	
Reduced opening hours of services/facilities	✓					✓	✓	✓		✓	✓										✓
Heterogeneity of services and practices			✓			✓			✓	✓							✓				
Fragmentation of care and lack of continuity of care	✓		✓	✓		✓				✓						✓		✓	✓		✓
Use of private services and relative economic impact	✓		✓	✓	✓	✓	✓	✓		✓	✓		✓	✓		✓		✓	✓		✓
Reduced support for GPs	✓		✓	✓	✓	✓		✓		✓	✓	✓	✓			✓		✓	✓		
Reduced HScPs training	✓		✓	✓		✓				✓									✓		
– Reduced staff training in non-specialist services (e.g. E.R, long-term care facilities)	✓	✓	✓	✓		✓	✓	✓	✓	✓	✓	✓	✓	✓			✓	✓	✓		
Reduced training of family assistants		✓		✓	✓		✓	✓				✓				✓	✓	✓		✓	
Ineffective communication	✓	✓	✓					✓	✓	✓					✓						✓
– Ineffective communication of diagnosis		✓	✓			✓			✓	✓					✓		✓	✓			

Long and complex diagnostic process				✓		✓													✓		
Riduced care of FmCs						✓			✓	✓	✓	✓	✓	✓		✓			✓		✓
− Feelings of abandonment and lack of psychological support	✓	✓	✓	✓		✓	✓	✓	✓	✓	✓	✓	✓	✓	✓	✓	✓	✓	✓	✓	✓
− Difficulties in coping with changes in daily life		✓											✓	✓				✓		✓	
Difficulties in managing medication	✓	✓	✓	✓	✓	✓							✓							✓	✓
Reduced non-pharmacological treatment options	✓	✓		✓		✓						✓	✓					✓			
Long and complex legal and bureaucratic aspects	✓		✓	✓	✓	✓			✓	✓			✓	✓	✓	✓	✓	✓		✓	✓
Ineffective information system	✓	✓	✓	✓		✓		✓	✓		✓	✓	✓	✓	✓	✓		✓	✓	✓	
Reduced sensitivity/empathy and stigma		✓			✓	✓	✓			✓	✓		✓			✓	✓	✓	✓		
**Improvements in care provision**																					
Functioning of territorial services		✓	✓								✓				✓						
− Increasing the number of DCCs		✓									✓			✓	✓			✓		✓	
Functioning of residential services				✓			✓		✓												
Availability and functioning of home care				✓	✓	✓	✓				✓						✓				✓
Greater involvement of GPs					✓						✓										
Better distribution and territorial increase of associations, AC and third sector initiatives	✓	✓	✓	✓	✓			✓		✓	✓	✓	✓						✓		
Networking between services and establishment of ICPs								✓	✓										✓		
Increased human resources dedicated to the care of PLWD	✓					✓					✓	✓	✓					✓	✓	✓	
Improvement of information services		✓	✓	✓		✓			✓	✓	✓	✓	✓	✓			✓	✓		✓	
Provision of non-pharmacological treatments											✓							✓			✓
Training of HScPs					✓	✓	✓		✓	✓	✓	✓									
Training of family assistants							✓	✓									✓				
Training of FmCs					✓						✓						✓				
FmCs support		✓				✓		✓	✓		✓	✓			✓			✓	✓	✓	
− Increased economic support					✓			✓								✓			✓		
Community awareness and creation of Dementia Friendly Communities							✓						✓								
Raising awareness of the condition within non-specialist services (e.g. E.R, long-term care facilities)				✓		✓		✓			✓	✓					✓				
Simplification of bureaucratic/administrative aspects				✓																	
**The impact of COVID-19 emergency**																					
Social isolation/need for socialisation					✓			✓			✓		✓				✓	✓			
Closure of services/interruption of activities		✓			✓			✓	✓	✓	✓	✓	✓	✓	✓		✓	✓	✓	✓	✓
− Delays in resuming activities		✓																✓	✓		
Reduction in visits to residential facilities		✓	✓	✓	✓					✓		✓				✓					
Aggravation of symptoms	✓	✓	✓		✓			✓			✓	✓	✓	✓	✓	✓	✓	✓	✓		✓
− Difficulties in managing the person at home and loss of routine		✓							✓			✓					✓		✓	✓	
Development of telecare/telemedicine					✓						✓							✓	✓		
− Usage difficulties									✓		✓										
Guaranteed services despite emergency	✓																				
**FmCs training needs**																					
How to welcome illness and deal with the sick person			✓							✓	✓	✓									

– Moments of confrontation, among peers and with professionals			✓							✓				✓		✓					
How to navigate between services			✓					✓		✓			✓	✓		✓		✓	✓		
The evolution of the disease and how to behave in the different phases	✓		✓	✓	✓	✓	✓	✓	✓	✓	✓		✓	✓		✓	✓	✓		✓	
Management of daily life (adaptation to the home environment, management of polypathologies, pharmacological treatment, communication aspects, etc.)	✓	✓	✓	✓	✓	✓	✓	✓	✓		✓	✓	✓	✓	✓	✓	✓	✓	✓	✓	✓
Emergency management				✓													✓				
Information on bureaucratic, legal and ethical aspects (e.g. advance care planning, legal figure nomination)					✓	✓			✓		✓			✓		✓					
